# PP52 Pharmaceutical Innovativeness Index – FDA-Approved Prostate Cancer Medications (2011 To 2021): A Case Study

**DOI:** 10.1017/S026646232400223X

**Published:** 2025-01-07

**Authors:** Ariane André, Ludmila Gargano, Isabela Freitas, Francisco de Assis Acurcio, Juliana Alvares-Teodoro, Augusto Guerra

## Abstract

**Introduction:**

Prostate cancer (PC) constitutes 13.5% of all cancer cases globally. Treatment is individualized and depends on various factors. In recent years, there has been an increase in the approval of drugs for the disease by the U.S. Food and Drug Administration (FDA). The study aimed to assess the innovativeness of FDA-approved drugs for PC treatment from 2011 to 2021.

**Methods:**

A search was conducted in the FDA database to identify medications approved from 2011 to 2021 and their supporting studies for approval. The assessment of the value of innovations was performed through the Pharmaceutical Innovativeness Index (PII), a methodology that quantitatively evaluates innovations in four domains: Therapeutic Need and Added Therapeutic Value of the new medication (graded on five levels from important to absent), and Study Design and Methodological Quality (graded on three levels) of the pivotal studies used as a data source for evaluation. Medications are assessed for a specific clinical indication and compared to available therapeutic alternatives.

**Results:**

Seven medications were identified for the treatment of PC, targeting different stages of the disease. The drugs were evaluated with a score ranging from 0 to 100, measuring the degree of innovativeness across the four assessed domains. Five (70%) medications scored above 50.0. The majority of the medications addressed a significant Therapeutic Need (n=4; 56%). The Added Therapeutic Value was assessed based on the survival gain compared to the comparator, with five medications considered poor (70%) and two moderate (30%). Regarding the quality of evidence, most studies showed a low risk of bias and a partially adequate design.

**Conclusions:**

In recent years, there has been an increase in the development of drugs for prostate cancer. However, the Added Therapeutic Value shows a small to moderate increase in survival compared to existing treatments. Many studies used a placebo instead of comparing new medications with available therapies. The PII indicates that, despite advancements, new technologies are needed to improve patient survival.

